# Correction: Therapeutic potential of flavonoids and rutin against inflammation-induced on a huntington animal model

**DOI:** 10.1371/journal.pone.0355871

**Published:** 2026-08-11

**Authors:** The PLOS One Staff

There are errors in the author affiliations. The correct affiliations are as follows:

David Calderón Guzmán^1^, Norma Osnaya Brizuela^1^, Maribel Ortiz Herrera^2^, Hugo Juárez Olguín^3^, Armando Valenzuela Peraza^1^, Alberto Rojas Ochoa^4^, Ernestina Hernández García^5^, Rafael Coria Jiménez^2^, Daniel Santamaria del Angel^1^

**1** Laboratorio de Neurociencias, Instituto Nacional de Pediatría (INP), Mexico City, Mexico, **2** Laboratorio de Bacteriología Experimental. INP, Mexico City, Mexico, **3** Independent Author, **4** Laboratorio de Oncología Experimental INP, Mexico City, Mexico, **5** Laboratorio de Farmacología, INP, Mexico City, Mexico

The publisher apologizes for the error.
